# Treatment of the Mamma during Childbed

**Published:** 1857-11

**Authors:** 

**Affiliations:** Stuttgardt


*Treatment of the Mamma during Childbed.* By Dr. Durr, of Stuttgardt.

There is hardly another therapeutic field in which the practitioner encounters so many faults and errors as in the management of the female breasts during childbed; it may not, therefore, be unprofitable to indulge in a few reflections in regard to it. There can be no doubt that nature has intended the mother to suckle her own child, but it is equally clear that she has denied her that privilege, either through imperfection of the nipple, or glandular structure (and consequent lack of milk) of the breast, in many instances. Disease, if it be not contagious, as syphilis, for instance, should not prevent the child from sucking, provided there is plenty of milk and a good nipple.

Notwithstanding the self-evident character of these principles, we see them violated daily. A mother has a flat, small nipple, that the child cannot seize—a simple negative to the act of sucking; the midwife, however, resorts to all sorts of appliances to induce the child to nurse, until it is exhausted and the breast inflamed; in short, until nature places her veto in an unmistakeable manner upon the proceedings. The same thing occurs when there is a paucity of milk. Breasts that at first sight convince us they are not intended to give milk in sufficient quantities for any useful purpose, merely rudimentary in size and form, are sucked and pumped until the dreadful pain to the mother makes them unendurable. The most of midwives, notwithstanding the severest pain and most obvious symptoms of inflammation, continue in the most persevering manner their efforts to get milk; the more tender, in fact, swollen and painful the breasts, the less milk the child gets; the more frequently the pump is applied.

Dr. Durr is never called to see a woman on account of inflamed breasts in childbed, but what he finds sucking glasses or other apparatus for drawing, and thinks that the physician should take every opportunity to earnestly condemn and forbid the use of them. The swelling of the breasts is taken as cakeing or clogging of milk in them, and an indication for greater efforts to get it out. When the pump draws no more milk, the the midwife tries to "bring it." Now it is warmed, smoked and cataplasmed, all with the same result; the inflammation, tenderness, and dryness (of milk) are increased, and finally followed by suppuration. And the abcess once formed, the same process is continued, until the whole gland is undermined, and one hole after another riddles the breast. Dr. Durr has met with the most happy success, in a large number of cases, by the following course of treatment: If the nipples are flat and small, impracticable, in fact, let them alone, and nourish the child with diluted cows' milk. Should the breasts be too small, or the mother so badly constituted, that we can predict her incapacity to afford milk enough for the child, no attempt to nurse should be made, and thus prevent the suffering that would inevitably flow from a trial. If the breasts are well developed and filled, and the nipple prehensible, let the child nurse, although the mother may be a sufferer, even phthisical. So soon, however, as pain, either in the nipple or breast, commences, at once desist from nursing, for it is an evidence either of a failure of milk, or the beginning of inflammation. Should one breast be inflamed, and the other not, the child should not be allowed to suck the sound breast, because the concert of function between the two organs will keep up excitement in the diseased one. If a breast is swollen and sensitive, all irritation should be kept from it; it should not be sucked, warmed or cataplasmed, for fear of suppuration and destruction of the milk tubes. On the other hand, resistance of the skin and integuments must be increased. The vessels of the breast must be compressed, and the stream of fluids be turned from the organ. Warmth cannot do this, cold may promote it. Dr. Durr has lately seen the most marked, speedy, good effects done from cold, in the case of a young, robust woman, with large, distended breasts, who had nursed several children before, but now was delivered of a dead child, and once before had abcess brought about by warm cataplasms. She dreaded the effect of the *ancient regime*, and in spite of the remonstrances of the midwife, made use of cold poultices. In twenty-four hours all swelling and sensitiveness had subsided, and the patient remained well until her next confinement, when, by similar means, she stove off an inflammation, and was enabled to nurse her child. But there are many puerperal patients who cannot use cold without bad effects,* and hence must command other resources, and they are very simple.

If the breast is only moderately inflamed, low diet, laxative medicines, and a light linen cover over the breast, is all that will be necessary to insure a subsidence of it and a free secretion of milk, and in four or five days the thing is all over.

In higher grades of inflammation, it will be necessary to give some mechanical support to the power of resistance against the afflux of blood to the part. For this purpose, some good plaster, applied to the whole mamma, leaving out the nipple, will do great good. The best article for this purpose is collodion, which, while it is light, makes firm and agreeable pressure on the whole organ. The collodion must be good, and applied thickly every hour or two until a consistent and thick shell is formed over the surface of the whole organ. Should we, however, on account of the price or smell of the collodion, desire to avoid its use, we may substitute adhesive plaster. It should be cut in a large square, with the corners rounded off, and a hole in the middle for the nipple. The diachelon, lead, black, soap, and Indian plasters all act in the same mechanical manner. Any plaster that produces irritation is injurious in their effects. The overflowing milk should be absorbed by clean dry linen, the breast suspended, and the arm kept at rest. If physicians will be contented with this simple plan of managing the breast, keep the bowels open, allow nothing but light diet, and avoid all irritation, one per cent of the breasts that are inflamed would not suppurate, and in four or five days the storm would all be over. If the breast has, either from neglect or irritating

* This I do not believe.—TRANS.

treatment, suppurated, the warmth and cataplasms should not be used, unless we desire to increase the pus, destroy more of the gland, and add very much to the suffering of our patient. We ought to satisfy ourselves by painting the formed but not opened abcess with diluted tinct. of iodine two or three times a-day, and cover it with a soft, light plaster. Under modified pain, the abcess will, in a short time, become so thinned as to break or permit lancing and evacuation of the matter.

The softening of the hardness left by the inflammation in the glands will best take place under careful covering of the breast, keeping up a constant laxative influence in the bowels, and avoiding all irritating diet.—*Allg. Med. Cent. Zeitung.*

